# The pharmacokinetics and metabolism of ifosfamide during bolus and infusional administration: a randomized cross-over study.

**DOI:** 10.1038/bjc.1998.161

**Published:** 1998-03

**Authors:** J. M. Singer, J. M. Hartley, C. Brennan, P. W. Nicholson, R. L. Souhami

**Affiliations:** Department of Oncology, University College, London, UK.

## Abstract

In a randomized cross-over trial, 11 patients received ifosfamide (IFOS) in 21-day cycles, which alternated between 3 g m(-2) x (2 or 3) days given as a 1-h bolus doses, or the same total dose as a continuous infusion. Patients who received four or more cycles also alternated between two cycles on dexamethasone 4 mg 8 hourly for 3 days starting 8 h before IFOS, and two cycles off dexamethasone. A total of 34 patient cycles were studied and serum and urinary levels of IFOS, 2 dechloroethylifosfamide (2DC), 3 dechloroethylifosfamide (3DC), carboxyifosfamide (CX) and isophosphoramide mustard (IPM) were measured by thin-layer chromatography. No significant differences could be detected in the areas under the curve (AUCs) of serum concentration, nor in the proportion of IFOS or its metabolites found in the urine. There was no significant effect of dexamethasone on IFOS metabolism. These results indicate that there is no identifiable pharmacokinetic basis for insistence on either bolus or infusional methods of IFOS administration.


British Journal of Cancer (1998) 77(6), 978–984
© 1998 Cancer Research Campaign

### The pharmacokinetics and metabolism of ifosfamide during bolus and infusional administration: a randomized cross-over study

**JM Singer, JM Hartley, C Brennan, PW Nicholson and RL Souhami**

Department of Oncology, University College, 91 Riding House St, London W1P 8BT, UK

**Summary** In a randomized cross-over trial, 11 patients received ifosfamide (IFOS) in 21-day cycles, which alternated between 3 g $m^{-2}$ × (2 or 3) days given as a 1-h bolus doses, or the same total dose as a continuous infusion. Patients who received four or more cycles also alternated between two cycles on dexamethasone 4 mg 8 hourly for 3 days starting 8 h before IFOS, and two cycles off dexamethasone. A total of 34 patient cycles were studied and serum and urinary levels of IFOS, 2 dechloroethylifosfamide (2DC), 3 dechloroethylifosfamide (3DC), carboxyifosfamide (CX) and isophosphoramide mustard (IPM) were measured by thin-layer chromatography. No significant differences could be detected in the areas under the curve (AUCs) of serum concentration, nor in the proportion of IFOS or its metabolites found in the urine. There was no significant effect of dexamethasone on IFOS metabolism. These results indicate that there is no identifiable pharmacokinetic basis for insistence on either bolus or infusional methods of IFOS administration.

**Keywords:** ifosfamide; pharmacokinetics; thin-layer chromatography; schedule; dexamethasone

The oxazaphosphorine, ifosfamide (IFOS) is used to treat a large number of malignancies (Brade et al, 1985). IFOS is a prodrug, requiring biotransformation for activity or toxicity (Allen and Creaven, 1972; Creaven et al, 1974). Metabolism appears to be mainly in the liver where cytochrome P450 enzymes produce the active intermediate 4 OH ifosfamide (Brock and Hohorst, 1963). This metabolite exists in equilibrium with its tautomer aldo-ifosfamide, which may be oxidized to carboxyifosfamide (CX) or decompose spontaneously, by β elimination to produce isophosphoramide mustard (IPM) (Brock, 1983). In addition to ring oxidation, side-chain oxidation at the exo- or endo-cyclic nitrogen may occur, yielding the metabolites 2- and 3-dechloroethyl ifosfamide (2DC), (3DC) and chloroacetaldehyde (CAA) (Figure 1).

IPM is the ultimate alkylating metabolite of IFOS, causing DNA interstrand cross-links. The metabolites responsible for nephrotoxicity and neurotoxicity are unknown, although CAA has been implicated as this is a reactive metabolite and produced in large quantities (Wagner, 1994). Concentrations of 2DC and 3DC are an indirect measurement of the unstable metabolite CAA, as this is formed in equimolar amounts in the *N*-dechloroethylation process.

A number of clinical pharmacokinetic studies have now been published showing considerable inter- and intra-individual variability in serum and urine levels of parent drug and metabolites (Creaven et al, 1974; Hartley et al, 1994; Boddy et al, 1993; 1995a; 1996). Some of this variability may be due to the differences in detection method (Nelson et al, 1976; Wagner et al, 1994), the schedule of administration, schedules, age (Lind et al, 1989*a*), the volume of distribution (Lind et al, 1989*b*), and the patient's age and hepatic and renal function. Metabolic factors may also be responsible such as genetic differences in the mixed function oxidases or drug interactions involving the cytochrome P450 systems (Lind et al, 1990; Kaijser et al, 1993; 1994; Kurowski & Wagner, 1993).

In standard IFOS doses (5–10 g $m^{-2}$ per cycle), many schedules of administration are used, such as a bolus dose in 30–60 min over 2–5 days and 24 or 72 h continuous infusions. It is unclear whether there is an optimal schedule, either for anti-tumour effect or for the reduction of toxicity, and whether the pharmacokinetics of IFOS and its principal metabolites are significantly influenced by schedule. Animal studies have shown that four fractionated doses of IFOS were superior to a single bolus in producing a higher tumour cell kill for less haematological and urothelial toxicity (Klein et al, 1984). Clinical studies are limited but also suggest that continuous infusion IFOS is haematologically less toxic than bolus doses for the same anti-tumour effect (Rodriguez et al, 1976; Morgan et al, 1982; Klein et al, 1984; Anderson et al, 1993). Thus, it is possible that increasing fractionation of IFOS may confer some sparing of normal tissue toxicity. In children, Boddy et al (1995*b*) have compared the pharmocokinetics of IFOS and metabolites under bolus and infusion; however, there have been no similar measurements in adults.

IFOS metabolism has been studied in vitro using rat and human liver microsomes (Ruzicka & Ruenitz, 1992; Murray et al, 1994; Walker et al, 1994). Weber and Waxman (1993) have shown that pretreatment of rats with dexamethasone (DEX) increased liver microsomal activation of ifosfamide sixfold. DEX is commonly used in combination with ifosfamide because of its antiemetic properties. It use may therefore alter the metabolic pattern of ifosfamide with therapeutic or toxic consequences.

The aims of this study were, therefore, to compare the pharmacokinetics of 1-h bolus dose vs continuous infusion IFOS in a

*Received 23 July 1996*
*Revised 18 August 1997*
*Accepted 28 August 1997*

*Correspondence to:* RL Souhami

**Figure 1** Metabolic pathways of ifosfamide randomized cross-over study and to study the effect of DEX administration on IFOS metabolism.

### METHODS

### Patient characteristics and sample collection

Patients with Ewings' sarcoma or soft tissue sarcoma were randomly assigned to receive IFOS as either a 1 h bolus dose or a continuous infusion for the first cycle and with or without DEX. On subsequent cycles, the patients were crossed-over to the alternative modes for both IFOS and DEX. Seven patients received IFOS at a dose of 3 g $m^{-2}$ × 2 days each cycle and four received IFOS 3 g $m^{-2}$ × 3 days each cycle. Bolus IFOS was administered as a rapid 1 h infusion. The dose of sodium-2-mercaptoethane-sulphonate (MESNA), the saline volume and rate were kept constant in each arm of the study. All patients were studied for 2–6 cycles and thus crossed-over at least once (Figure 2). A total of 34 patient/cycles were studied.

Eleven patients were studied, aged 23–71 years (four men and seven women). One patient required a 15% dose reduction of IFOS after the first cycle because of bone marrow toxicity. All other patients received 100% of the prescribed dose for each cycle studied. Other co-administered drugs were: doxorubicin (11 patients), vincristine (one patient), paracetamol (four patients) lorazepam (three patients) and metaclopramide (11 patients). Blood samples were drawn immediately before treatment and at 2 to 3 h intervals throughout the 3-day period, except for 8 h night intervals. Samples were centrifuged immediately at 3000 r.p.m. at 4°C, serum separated and stored at –20°C for subsequent analysis. Urine samples were collected immediately before treatment and in 6 hourly time blocks for the 3-day period. Urine samples were frozen immediately at –20°C for analysis.

### Sample preparation and TLC

The parent drug, ifosfamide (IFOS), and its metabolites (2-chloroethyl)-2-amino-tetrahydro-2-oxide-2H-1, 3, 2-oxazaphosphorine (2 dechloro- ethylifosfamide; 2DC), 2-(2-chloro-ethyl)-amino-tetrahydro-2-oxide-2H-1,3,2-oxazaphosphorine (3-dechloroethylifosfamide; 3DC), 3-[*N*, *N'*-bis(2-chloroethyl-amino) phosphinyloxy] propanic acid (carboxyifosfamide; CX) and *N*, *N'*-bis (2-chloroethyl) phosphorodiamidic acid (isophosphoramide mustard; IPM) were all prepared, authenticated and kindly given by Asta Medica (Frankfurt, Germany).

© Cancer Research Campaign 1998 

**Figure 2** Treatment schedules for bolus (upper) and infusion (lower) administrations

Extraction of parent drug and metabolites from patient urine was as described previously (Boddy and Idle, 1992; Hartley et al, 1994). Samples were applied to prewashed XAD-2 columns and eluted with methanol, dried and reconstituted in a small volume of methanol. An aliquot (100 mg) of silicic acid and 3 ml of methanol were added to the residue, vortexed and then centrifuged at 4000 r.p.m. for 10 min. The supernatant was collected and evaporated to dryness. Serum samples were treated similarly, but the XAD-2 columns were washed with 0.05 M Tris buffer pH 5.5 before sample application.

Samples were then reconstituted in 70 μl of methanol and 50 μl was applied to high-performance thin-layer chromatography (HPTLC) plates using a 'Linomat IV' TLC sample applicator (Camag, Germany). The TLC plates were placed in glass tanks containing dichloromethane-dimethylformamide-glacial acetic acid (90:7:1) and allowed to run the full height of the plate twice to ensure good resolution of all metabolites. After drying, the plates were placed in a second mobile phase of chloroform–methanol–glacial acetic acid (9:6:1) and allowed to run to a height of 3.0 cm twice. Once dry, the plates were then sprayed with 5% 4-(4-nitrobenzyl)pyridine (NBP) in acetone/0.2 M acetate buffer pH 4.6 (8:2, v/v) twice, heated at 150°C for 10–15 min and then dipped in 3% potassium hydroxide in methanol to develop the plates. Immediately after development, the TLC plates were scanned using a reflecting laser densitometer. With the exception of serum IPM, clean and reproducible chromatograms were obtained.

Concentrations were calculated using known standards of each metabolite and urine and serum controls. Although IPM was usually visible on the chromatograms, the recovery was poor. As a consequence, the values of the serum IPM levels were regarded as too unreliable and were disregarded. The recovery of serum IFOS, 3DC, 2DC and CX, all at 80 μm, from the internal controls was 95%, 80%, 60% and 40% respectively. The recoveries for IFOS, 3DC, 2DC, CX and IPM, all at 80 μm, in urine were 93%, 80%, 85%, 45% and 25% respectively. The coefficient of variation of the urinary assays was previously reported to be 3.2%, 5%, 7%, 13% and 32% respectively (Hartley et al, 1984) and for the serum assays for IFOS, 3DC, 2DC and CX were 22%, 23%, 21% and 12% respectively.

### Pharmacokinetic analyses of urinary and serum concentrations

Urinary collections were made over consecutive 6-h periods. No urinary drug or metabolite could be detected in the collections after 60 h from the start of administration, and so, for each cycle, the total urinary amounts of drug and metabolites over the first 60 h were calculated. These were then expressed as the molar fractions of the administered dose, and are referred to hereafter as a *urinary fraction* of dose.

**Figure 3** Serum concentration of IFOS and three metabolites in a patient under bolus (**A**) and continuous infusion (**B**) administration. ▣, IFOS; ◆; ZDC; ■, 3DC; ◇, CX

For the serum determinations the area under the curve ($AUC_\infty$) from time zero to infinity of the concentration vs time was assessed primarily from the experimental data points by the linear trapezoidal rule. In those cases in which the concentration of the last sample had not reached the baseline, it was necessary to estimate the area in the tail portion. This was based on extrapolation of the last few data points using an exponential decay curve. To adjust

© Cancer Research Campaign 1998

**Table 1** Mean and s.d. of peak serum concentrations (μM) of IFOS and its metabolites aggregated over all patients and treatment cycles (total of *n* cycles) and categorized according to mode of administration (A) or by absence/presence of co-administration of dexamethazone (B). The *P* values are those relating to within patient comparisons from an ANOVA (see text)

**A**

| | Bolus dose | | | Continuous infusion | | | *P*-value |
|---|---|---|---|---|---|---|---|
| | Mean | s.d. | *n* | Mean | s.d. | *n* | |
| IFOS | 380.1 | 157.2 | 14 | 185.4 | 109.7 | 13 | 0.002 |
| 2DC | 52.7 | 52.5 | 9 | 26.3 | 21.0 | 12 | >0.05 |
| 3DC | 104.9 | 39.9 | 10 | 92.5 | 97.3 | 12 | >0.05 |
| CX | 69.1 | 34.2 | 10 | 56.4 | 55.6 | 10 | >0.05 |

**B**

| | Dexamethasone | | | No dexamethasone | | | *P*-value |
|---|---|---|---|---|---|---|---|
| | Mean | s.d. | *n* | Mean | s.d. | *n* | |
| IFOS | 268.9 | 136.4 | 15 | 308.2 | 202.4 | 12 | >0.05 |
| 2DC | 44.2 | 50.8 | 11 | 30.3 | 19.8 | 10 | >0.05 |
| 3DC | 111.0 | 97.9 | 12 | 82.7 | 32.6 | 10 | >0.05 |
| CX | 71.3 | 63.9 | 9 | 55.8 | 23.2 | 11 | >0.05 |

**Table 2** Mean and s.d. of AUC (mM h) of IFOS and its metabolites aggregated over all patients and treatment cycles (total of *n* cycles) and categorized according to mode of administration (A) or by absence/presence of co-administration of dexamethazone (B)

**A**

| | Bolus dose | | | Continuous infusion | | |
|---|---|---|---|---|---|---|
| | Mean | s.d. | *n* | Mean | s.d. | *n* |
| IFOS | 6.89 | 2.03 | 14 | 6.96 | 4.40 | 13 |
| 2DC | 0.98 | 1.27 | 9 | 0.64 | 0.44 | 12 |
| 3DC | 3.14 | 2.01 | 10 | 2.92 | 2.73 | 12 |
| CX | 0.91 | 0.55 | 10 | 1.52 | 2.36 | 10 |

**B**

| | Dexamethasone | | | No dexamethasone | | |
|---|---|---|---|---|---|---|
| | Mean | s.d. | *n* | Mean | s.d. | *n* |
| IFOS | 7.20 | 4.10 | 15 | 6.57 | 2.09 | 12 |
| 2DC | 1.04 | 1.12 | 11 | 0.50 | 0.41 | 10 |
| 3DC | 3.33 | 3.00 | 12 | 2.65 | 1.38 | 10 |
| CX | 1.57 | 2.51 | 9 | 0.92 | 0.48 | 11 |

for minor differences in dose, all values of AUC and $C_{max}$, the peak concentration, were normalized to correspond to a total dose of 11.54 g, the mean value over all patients and cycles.

### Statistical methods

Analyses of variance (ANOVA) were made using the SPSS statistical package (SPSS, Chicago, IL, USA). As observations were generally not available for all combinations of the factors of interest (for example patient number, bolus/infusion administration, cycle number, use/absence of dexamethasone), it was only possible to test for the main effects. In all analyses the first of these factors, patient number, was always included in the ANOVA model. With the cross-over design of the investigation, this allowed for the testing for any effects due to any of the remaining three factors on a within-patient basis. Effects were regarded as statistically significant for $P < 0.05$.

**Figure 4** Scattergram with overall mean and s.e. of the area under the curve (AUC) of the serum concentration of ifosfamide and metabolites for all cycles of bolus and infusion administrations

### RESULTS

Eleven patients were studied over a total of 34 cycles of treatment. Of these, two patients declined venesection and one had insufficient urine collection. Seven patients were treated with IFOS over 48 h and four patients were treated with IFOS over 72 h.

### Serum levels

Figure 3 shows typical measurements of serum concentration of IFOS and metabolites, measured in the infusion and the bolus treatments of a patient receiving IFOS over 48 h. The concentration profile of IFOS shows an immediate rise after the bolus administration and a gradual rise after infusional administration. There are minor differences in the profiles of the metabolites in the example in Figure 3, but these were not seen systematically in the rest of the dataset. In general, comparing the two types of administration, the serum metabolite profiles were quite similar in both shape and magnitude.

### Summary values

Summary values of $C_{max}$ for all patients and cycles under the two administration modes, and with and without DEX are listed in Table 1. The ANOVA showed no effect due to DEX but the effect of the administration mode was significant for IFOS ($F$ = 13.3; d.f. 1,13; $P$ = 0.002) but not for any of the three metabolites. In summary, therefore, for the peak serum concentrations of IFOS and its metabolites, only those of IFOS were significantly different (by a factor of 2.05) in the bolus dose against the infusion.

© Cancer Research Campaign 1998

**Table 3** Mean and s.d. of urinary fraction (%) of IFOS and its metabolites aggregated over all patients and treatment cycles (total of *n* cycles) and categorized according to mode of administration (A) or by absence/presence of co-administration of dexamethazone (B). The *P*-values are those relating to within patient comparisons from an ANOVA (see text)

**A**

| | Bolus dose | | | Continuous infusion | | | *P*-value |
|---|---|---|---|---|---|---|---|
| | Mean | s.d. | *n* | Mean | s.d. | *n* | |
| IFOS | 21.0 | 9.0 | 16 | 25.2 | 14.5 | 15 | >0.05 |
| 2DC | 5.3 | 2.1 | 16 | 5.6 | 3.5 | 15 | >0.05 |
| 3DC | 12.4 | 4.9 | 16 | 12.2 | 6.1 | 15 | >0.05 |
| CX | 16.1 | 6.8 | 16 | 13.5 | 8.7 | 15 | >0.05 |
| IPM | 10.2 | 6.9 | 16 | 9.5 | 3.7 | 15 | >0.05 |

**B**

| | Dexamethasone | | | No dexamethasone | | | *P*-value |
|---|---|---|---|---|---|---|---|
| | Mean | s.d. | *n* | Mean | s.d. | *n* | |
| IFOS | 20.9 | 6.4 | 18 | 26.0 | 16.9 | 13 | >0.05 |
| 2DC | 5.1 | 2.5 | 18 | 5.9 | 3.2 | 13 | >0.05 |
| 3DC | 11.8 | 5.8 | 18 | 13.0 | 5.0 | 13 | >0.05 |
| CX | 15.8 | 6.3 | 18 | 13.5 | 9.6 | 13 | >0.05 |
| IPM | 10.5 | 3.8 | 18 | 9.1 | 7.4 | 13 | >0.05 |

**Table 4** Urinary fraction of dose as a percentage: grand mean and within-patient standard deviations

| | Grand mean | Within-patient s.d. |
|---|---|---|
| IFOS | 23.0 | 11.1 |
| 2DC | 5.4 | 2.3 |
| 3DC | 12.3 | 4.6 |
| CX | 14.8 | 7.2 |
| IPM | 9.9 | 4.8 |
| Total | 66.4 | 20.6 |

A preliminary analysis was made on the AUC of those treatment cycles consisting of a pair of bolus doses at a 24 h interval. Owing to the rapid decay of the concentration of IFOS after the first dose, it was possible to resolve the AUC into components contributed by each of the two doses. In ten treatment cycles in six patients, the AUC components contributed by the first and second dose had means of 4.96 mM h and 2.56 mM h respectively. A paired *t*-test showed that these were significantly different ($t = 3.33$, d.f. = 9, $P = 0.009$) and this seems to give clear evidence of induction of IFOS metabolism. It was not possible to apply this type of analysis to the AUCs of the metabolites as it was impossible to resolve the AUC into the components contributed by the two doses.

**Figure 5** Scattergram with overall mean and s.e. of the urinary fraction of dose (as percentage) for ifosfamide and metabolites for all cycles of bolus and infusion administrations

*© Cancer Research Campaign 1998*

Subsequent analyses were made in terms of the total AUC over the treatment cycle. Figure 4 gives a scatter plot of AUCs taken over all patients and cycles for bolus and infusion administrations, and Table 2 gives corresponding summary data. ANOVA of these with DEX, administration mode and cycle number as factors, either singly or in combination did not reveal any significant effects.

### Urine analysis

For each cycle the total urinary amounts of IFOS and metabolites were expressed as molar fractions of the amount of IFOS given. Patients receiving 72 h treatments were also included in this part of the analysis, giving a total of 31 cycles in ten patients. Figure 5 gives a scatter plot of these values, for bolus and infusion administration, and Table 3 gives corresponding summary data. The ANOVA did not reveal any significant differences on the urinary fractions of parent drug or metabolite of bolus/infusion administration or of absence/presence of DEX co-administration. The effect of sequential cycles was also examined, but the analysis did not reveal significant alteration in the proportion of metabolite generated with successive cycles. Thus, it appeared unlikely that there was induction of IFOS metabolism between successive cycles.

As the patients underwent more than one cycle of treatment, it was possible to derive from the ANOVA an estimate of the within-patient variability (i.e. from cycle to cycle) of the urinary fractions. For this, all urinary results were pooled (as no significant differences due to administration of DEX had been detected) and the values of the within-patient s.d. given in Table 4. These results reveal a considerable degree of within-patient variability in urinary excretion from cycle to cycle.

### DISCUSSION

Ifosfamide is commonly administered, either as a series of intravenous boluses over several days or as an infusion, yet there has been no direct pharmacokinetic comparison between the two schedules. Klein et al (1984) reported a study in which rats were treated with either a single or four bolus doses and demonstrated that the fractionated IFOS regimen resulted in a decreased $LD_{50/30}$ and higher tumour cell kill. Phase I/II clinical studies also indicated that fractionating the dose resulted in less microscopic and macroscopic haematuria (Rodriguez et al, 1976; Klein et al, 1984). More recently, Anderson et al (1993) randomized 159 patients with small-cell lung cancer to receive 5 g $m^{-2}$ IFOS, either over 24 h or 7 days with an ambulatory pump infusion, and found the survival to be the same but the haematological toxicity to be less in the 7-day infusion arm.

Previous pharmacokinetic studies have been reviewed recently by Wagner (1994) and Kaijser et al (1994). A number of studies were performed on patients who received either infusional or bolus IFOS, but which measured only the parent drug (Creaven et al, 1974; Allen and Creaven, 1975; Nelson et al, 1976). Of the reports that have made measurements on both IFOS and its principal metabolites, Kurowski and Wagner (1993) studied 11 patients receiving five daily boluses of IFOS and measured IFOS, 2DC, 3DC, and CAA and the unstable 4 hydroxyifosfamide. Other groups (Boddy and Idle, 1992; Boddy et al, 1993; 1995*a*; Hartley et al, 1994) have measured both IFOS and its principal metabolites in patients receiving infusional IFOS. In general, these studies have shown that there is induction of IFOS metabolism after approximately 3 days of treatment. With repeated daily bolus doses over 5 days, Lind et al (1989) reported a decrease in half-life that forms a mean of 6.2 h on the first day to 3.8 h on the fifth day and that this decrease was almost entirely explained by an increase in clearance.

In the example shown (Figure 3) of the profiles of each metabolite, there are some minor differences between that 1 h bolus and infusion administrations. However, over the whole dataset little systematic difference could be discerned, and cross-over comparison between 1 h bolus and infusional IFOS did not reveal a significant difference in the AUC. This is compatible with the cross-over study in 17 children reported by Boddy et al (1995*b*). Any alteration in the relative amounts of metabolites produced may be of therapeutic advantage. Less dechloroethylation would result in a reduction of 2DC, 3DC and CAA and, possibly, less toxicity. From our data, it appears that with bolus administration the metabolites are sufficiently slow to appear in the serum their concentration profiles are essentially no different from that with infusion administration. Thus, any differences in therapeutic or toxic effect of the two administration regimes do not appear to be reflected in differences in serum pharmacokinetics of these metabolites.

In those patients receiving cycles of two bolus doses, the AUC attributable to the second dose was less than that of the first. Although this was not the primary purpose of this investigation and the result was based on small numbers, it offers some evidence of induction and it parallels the findings of Lind et al (1989) referred to above.

In the second part of the study, we examined the effect of DEX on the metabolism of IFOS. Weber and Waxman (1993) showed that, in rats, phenobarbital induced cytochrome P450 subfamily 2B1 for the metabolism of both IFOS and cyclophosphamide. They also reported that DEX induced cytochrome P450 3A to increase the metabolism of IFOS sixfold (but not that of cyclophosphamide) and suggested the co-administration of DEX clinically may enhance response. Walker et al (1994) have suggested that the same enzyme is responsible for both IFOS activation and dechloroethylation in humans. In our study, patients were crossed over from no DEX to DEX 4 mg t.d.s. for 3 days, the latter being chosen as that in routine clinical use in conjunction with antiemetics. No difference in any of the pharmacokinetic parameters could be detected and so, at these dose levels of DEX, changes in the measured IFOS metabolites do not appear important. This is of clinical interest as DEX is widely used as an antiemetic for cytotoxic chemotherapy. For cyclophosphamide Yule et al (1995) reported an association of DEX pretreatment in children with the presence of serum ketocyclophosphamide. In our patients the levels of ketoifosfamide were barely detectable by the TLC technique and we were unable to confirm or deny any such similar association.

Several previous reports have drawn attention to the wide variability in IFOS handling between patients, and this is the case in our patients. However, it is apparent from our results (Table 4) that there is also a large degree of within-patient variability, i.e. from cycle to cycle. The total fraction of dose recovered in urine, for example, has a within-patient s.d. that corresponds to a coefficient of variation of 31%. Only a small part of this variability can be attributed to uncertainties in the experimental technique. The coefficient of variation of this assay has been reported previously by this group and for IFOS was found to be 4% (Hartley et al, 1994). Boddy et al (1996) recently reported a high degree of within-patient variability in ifosfamide pharmacokinetics in children. In

© Cancer Research Campaign 1998

11 patients the within-patient variation from cycle to cycle in serum AUC of drug was twofold and that of metabolites was up to tenfold. The large variability from cycle to cycle may mean that differences due to saturation or induction, stemming from mode of administration, or of the co-administration of DEX, may not be revealed, even in cross-over studies such as that reported here. Indeed, it could be argued that any such differences would be relatively unimportant clinically compared with the inherent variability of drug handling within a patient.

We conclude that, against this pattern of variability in drug handling in patients from cycle to cycle, we can detect no consistent significant differences in the serum pharmacokinetics, nor the urinary excretion, of IFOS and its metabolites, when under bolus as against infusion administration. From the pattern of serum pharmacokinetics and urinary excretion in adults, we can find no reason to prefer one regimen over the other in the clinical use of the drug. This is broadly in line with the conclusions of Boddy et al (1995*b*) for children.

### ACKNOWLEDGEMENT

This work was supported by a grant from the Special Trustees of the Middlesex Hospital.

### REFERENCES

Allen LM and Creaven PJ (1972) In vitro activation of isophosphamide (NSC-109724), a new oxazaphosphorine, by rat liver microsomes. *Cancer Chemoth Rep* **56**: 603–610

Allen LM and Creaven PJ (1975) Human pharmacokinetic model for isophosphamide (NSC-109724). *Cancer Chemother Rep* **59**: 877–882

Anderson H, Hopwood P, Prendiville J, Radford JA, Thatcher N and Ashcroft L (1993) A randomised study of bolus vs continuous pump infusion of ifosfamide and doxorubicin with oral etoposide for small cell lung cancer. *Br J Cancer* **67**: 1385–1390

Boddy AV and Idle JR (1992) Combined thin-layer chromatography-photography-densitometry for the quantification of ifosfamide and its principal metabolites in urine, cerebrospinal fluid and plasma. *J Chromatogr* **575**: 137–142

Boddy AV, Yule SM, Wyllie R, Price L, Pearson ADJ and Idle JR (1993) Pharmacokinetics and metabolism of ifosfamide administered as a continuous infusion in children. *Cancer Res* **53**: 3758–3764

Boddy AV, Proctor M, Simmonds D, Lind MJ and Idle JR (1995*a*). Pharmacokinetics, metabolism and clinical effect of ifosfamide in breast cancer patients. *Eur J Cancer* **31A**: 69–71

Boddy AV, Yule SM, Wyllie R, Price L, Pearson ADJ and Idle JR (1995*b*) Comparison of continuous infusion and bolus administration of ifosfamide in children. *Eur J Cancer* **31A**: 785–790

Boddy AV, Yule SM, Wyllie R, Price L, Idle JR (1996) Intrasubject variation in children of ifosfamide pharmacokinetics and metabolism during repeated administration. *Cancer Chemother Pharmacol* **32**: 147–154

Brade WP, Herdrich K and Varini M (1985) Ifosfamide – pharmacology, safety and therapeutic potential. *Cancer Treat Rev* **12** (suppl. A): 1–47

Brock N (1983) The oxazaphosphorines. *Cancer Treat Rev* **10**: 3–15

Brock N and Hohorst HJ (1963) Uber die Aktivierung von cyclophosphamid in vivo und in vitro. *Arzneim Forsch* **13**: 1021–1031

Creaven PJ, Allen LM, Alford DA and Cohen MH (1974) Clinical pharmacology of isophosphamide. *Clin Pharmacol Ther* **16**: 77–86

Hartley JM, Hansen L, Harland SJ, Nicholson PW, Pasini F and Souhami RL (1994) Metabolism of ifosfamide during a 3 day infusion. *Br J Cancer* **69**: 931–936

Kaijser GP, Korst A, Beijnen JH, Bult A and Underberg WJM (1993) The analysis of ifosfamide and its metabolites (review). *Anticancer Res* **13**: 1311–1324

Kaijser GP, Beijnen JH, Bult A and Underberg WJM (1994) Ifosfamide metabolism and pharmacokinetics (review). *Anticancer Res* **14**: 517–532

Klein OH, Wickramanyake PD, Christian E and Coerper C (1984) Therapeutic effects of single push or fractionated injections or continuous infusions of oxazaphosphorines (cyclophosphamide, ifosfamide, ASTA Z7557). *Cancer* **54**: 1193–1203

Kurowski V and Wagner T (1993) Comparative pharmacokinetics of ifosfamide, 4-hydroxyifosfamide, chloroacetaldehyde, and 2- and 3-dechloroethylifosfamide in patients on fractionated intravenous ifosfamide therapy. *Cancer Chemother Pharmacol* **33**: 36–42

Lind MJ, Margison JM, Cerny T, Thatcher N and Wilkinson PM (1989*a*). Comparative pharmacokinetics and alkyating activity of fractionated intravenous and oral ifosfamide in patients with bronchogenic carcinoma. *Cancer Res* **49**: 753–757

Lind MJ, Margison JM, Cerny T, Thatcher N and Wilkinson PM (1989*b*). Prolongation of ifosfamide elimination half-life in obese patients due to altered drug distribution. *Cancer Chemother Pharmacol* **25**: 139–142

Lind MJ, Roberts HL, Thatcher N and Idle JR (1990) The effect of route of administration and fractionation of dose on the metabolism of ifosfamide. *Cancer Chemother Pharmacol* **26**: 105–111

Morgan LR, Harrison JE, Hawke JE, Hunter HL, Costanzi JJ, Plotkin D, Tucker WG and Worrall PM (1982) Toxicity of single- vs. fractionated-dose ifosfamide in non-small cell lung cancer: a multi-center study. *Sem Oncol* **9**: 66–70

Murray M, Butler AM and Stupans I (1994). Competitive inhibition of human liver microsomal cytochrome P450-dependent steroid 6β-hydroxylation activity by cyclophosphamide and ifosfamide in vitro. *J Pharmacol Exp Ther* **270**: 645–649

Nelson RL, Allen LM and Creaven PJ (1976) Pharmacokinetics of divided-dose ifosfamide. *Clin Pharmacol Ther* **19**: 365–370

Rodriguez V, Bodley GP, Freireich EJ, McCredie KB, McKelvey EM and Tashima CK (1976) Reduction of ifosfamide toxicity using dose fractionation. *Cancer Res* **36**: 2945–2948

Ruzicka JA and Ruenitz PC (1992) Cytochrome P-450-mediated N-dechloroethylation of cyclophosphamide and ifosfamide in the rat. *Drug Metab* **20**: 770–772

Wagner T (1994) Ifosfamide clinical pharmacokinetics. *Clin Pharm* **26**: 439–456

Walker D, Flinois J-P, Monkman SC, Beloc C, Boddy AV, Cholerton S, Daly AK, Lind MJ, Pearson, ADJ, Beaune PH and Idle JR (1994) Identification of the major human hepatic cytochrome P450 involved in activation and n-dechloroethylation of Ifosfamide. *Biochem Pharmacol* **47**: 1157–1163

Weber GF and Waxman DJ (1993) Activation of the anti-cancer drug ifosfamide by rat liver microsomal P450 enzymes. *Biochem Pharmacol* **45**: 1685–1694

Yule SM, Boddy AV, Cole M, Price L, Wyllie R, Tasso MJ, Pearson DJ and Idle JR (1995) Cyclophosphamide metabolism in children. *Cancer Res* **55**: 803–809

© Cancer Research Campaign 1998


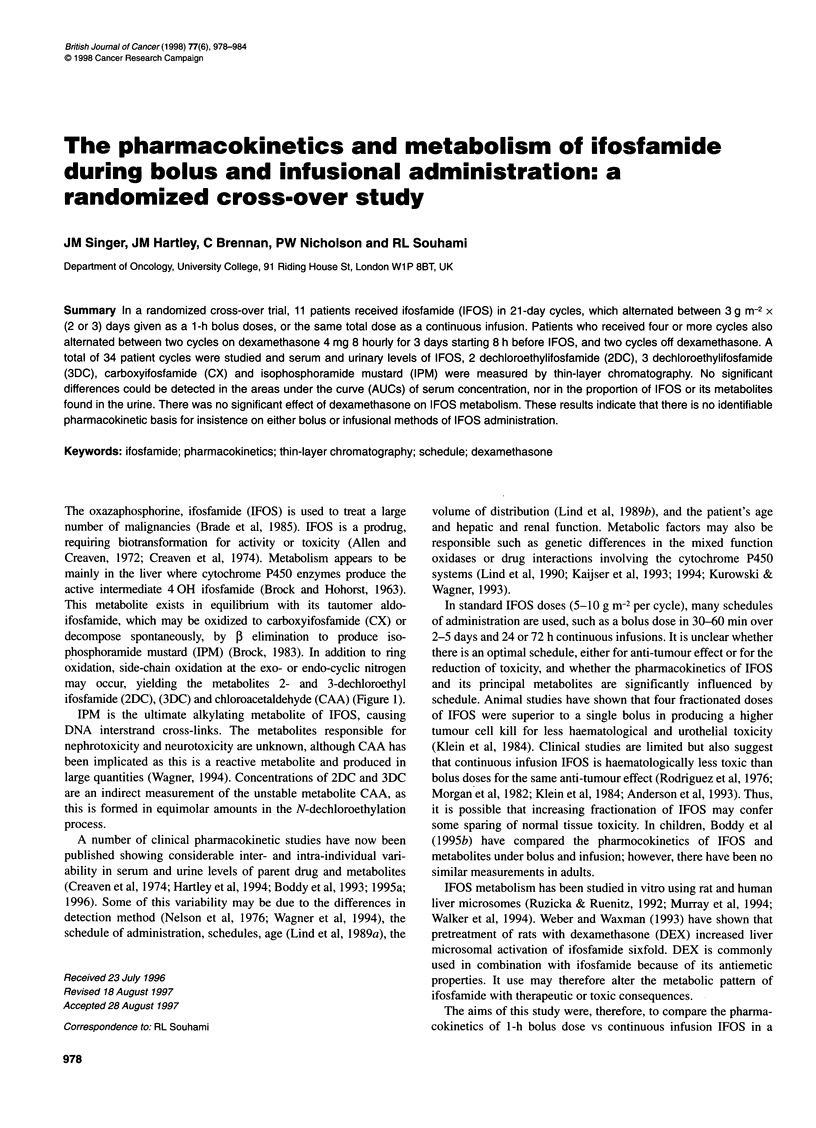

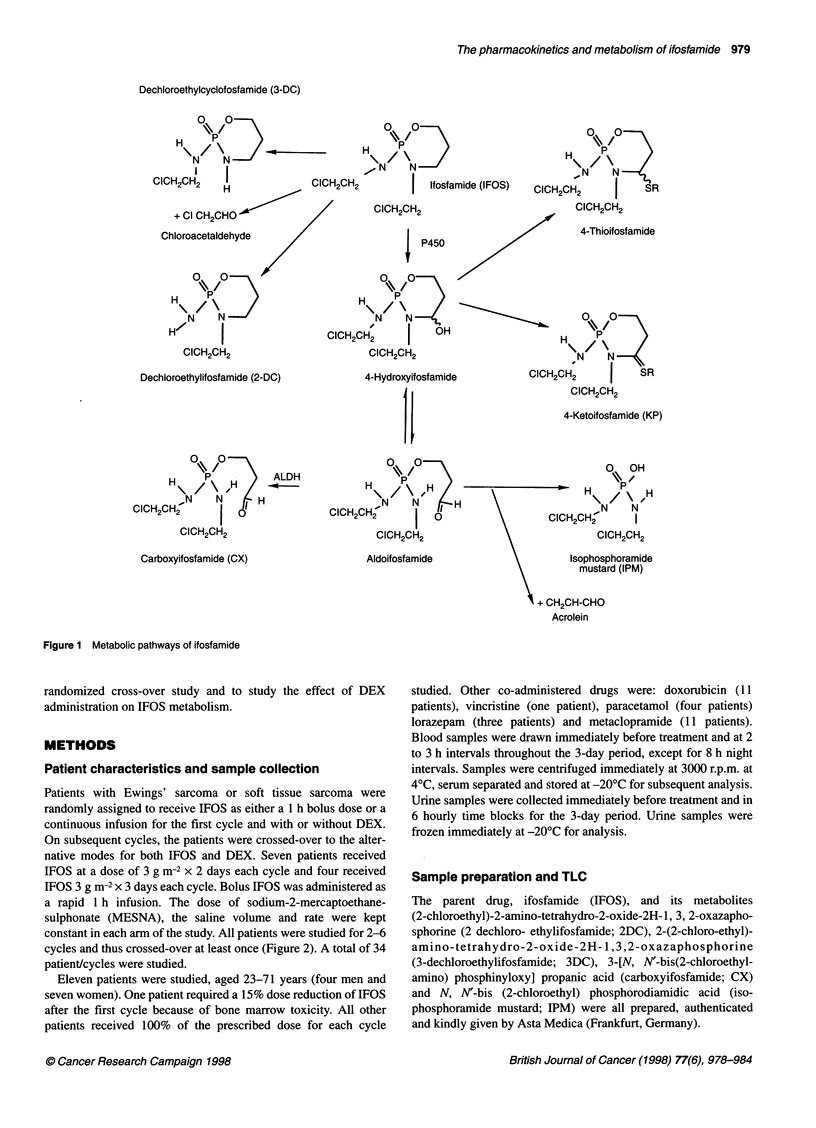

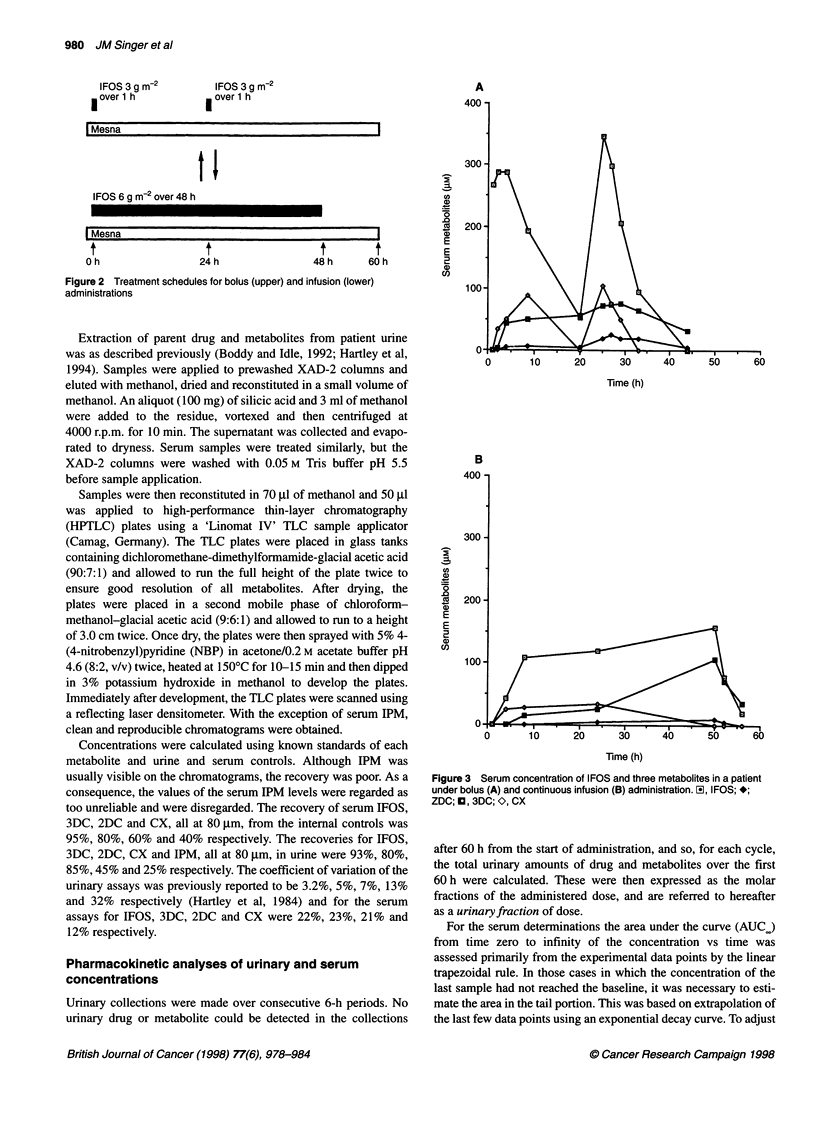

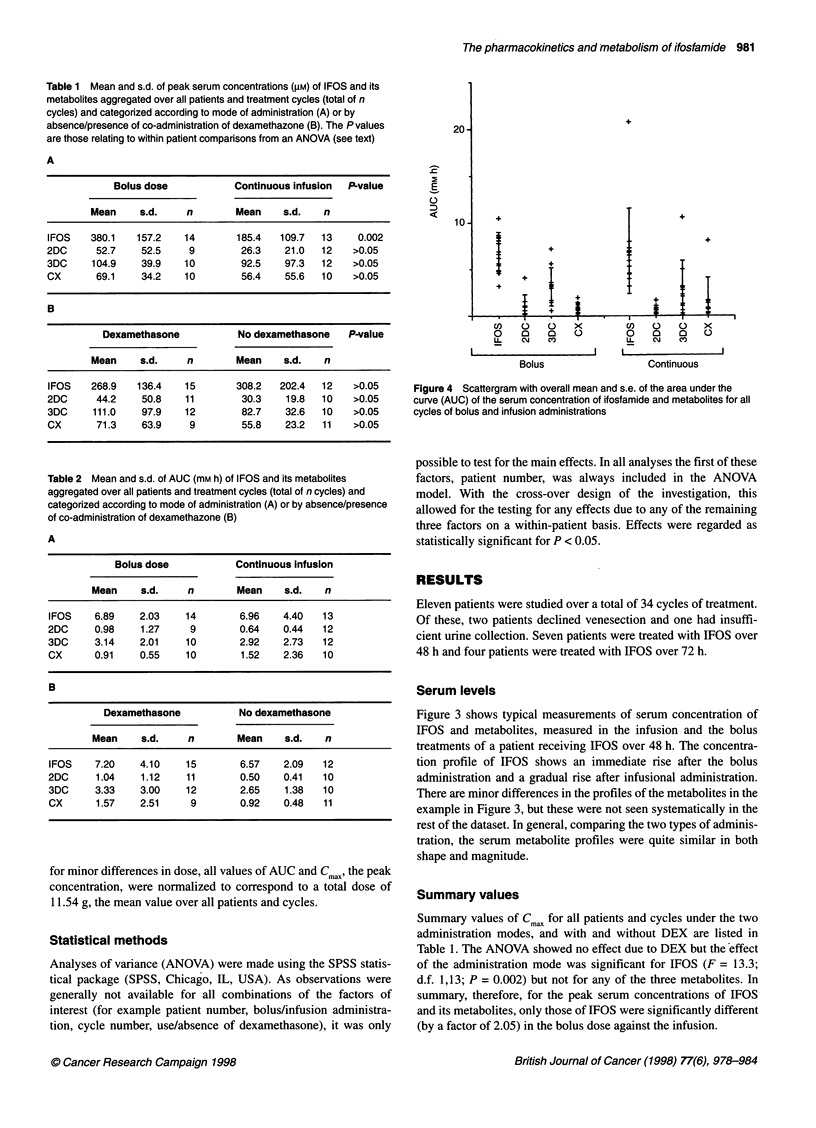

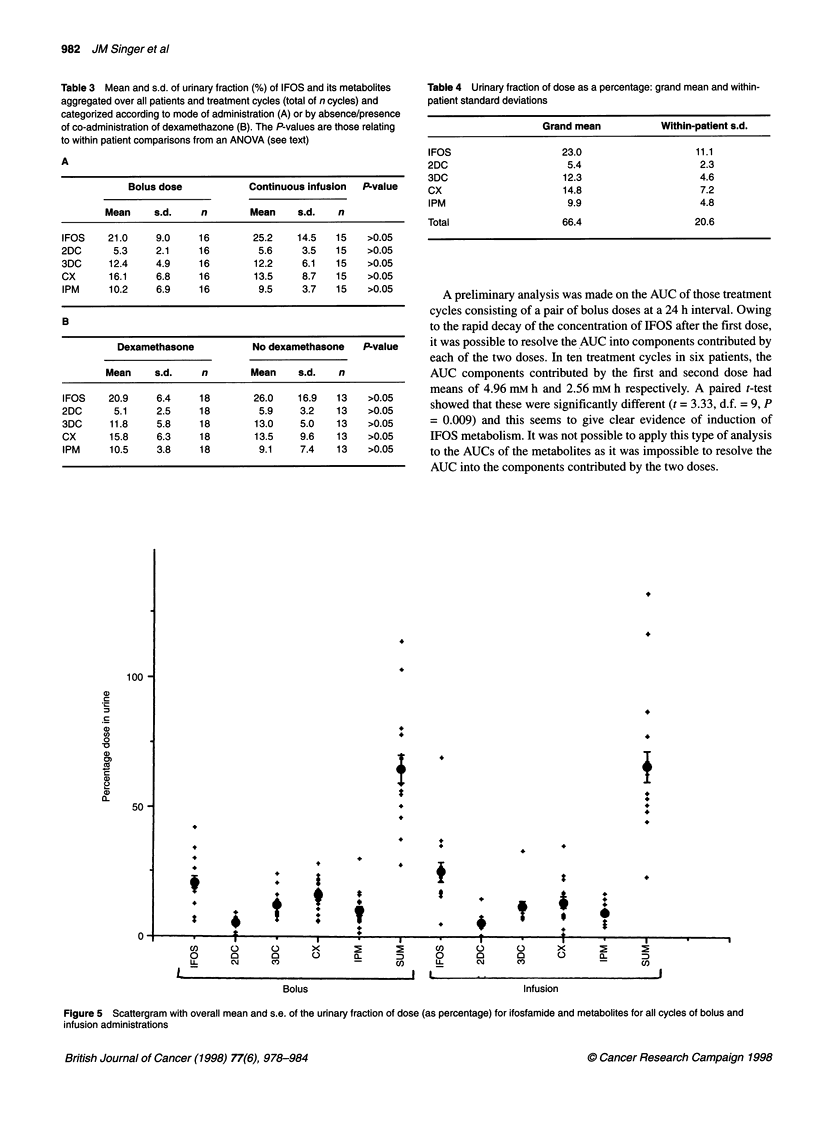

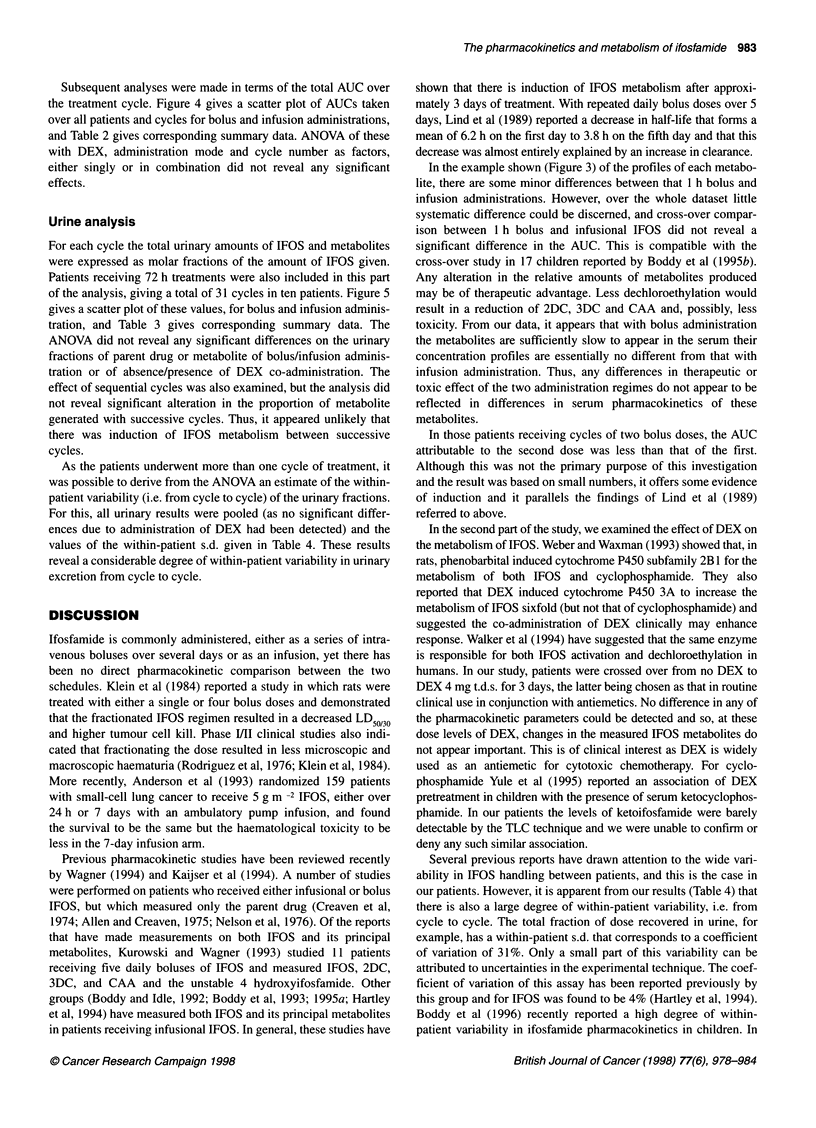

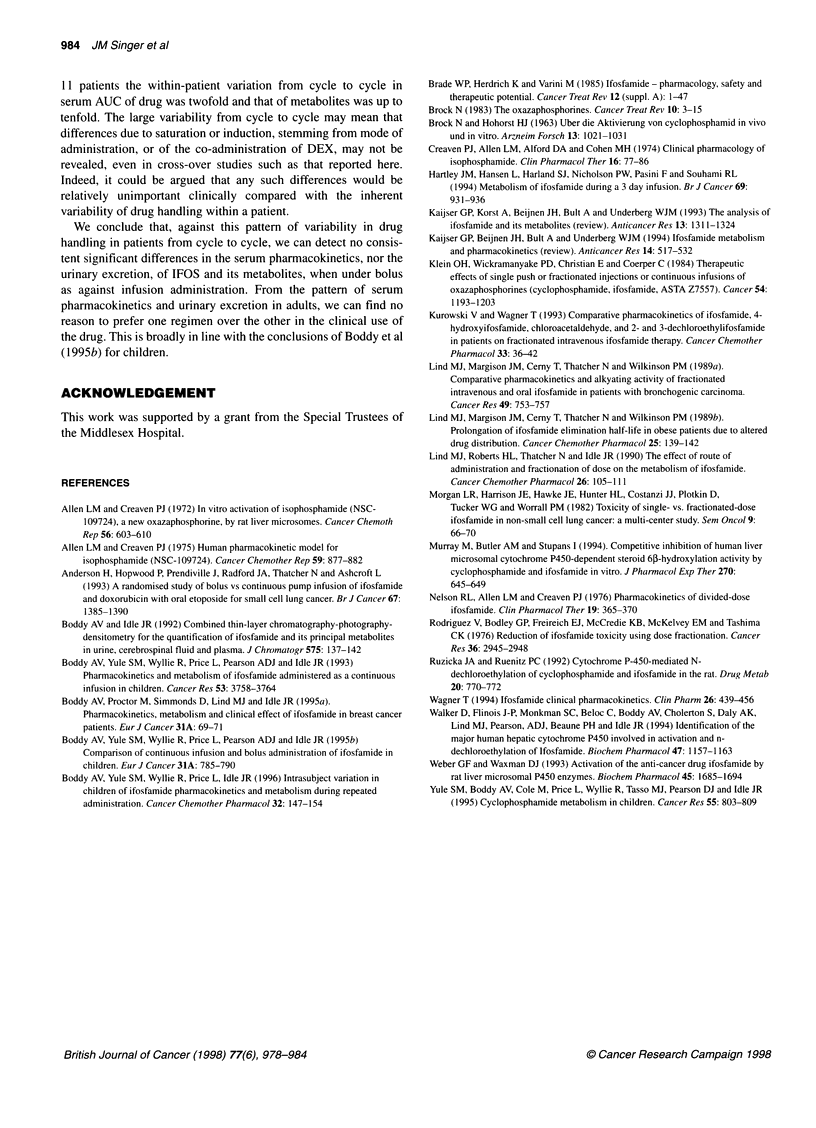


## References

[OCR_00839] Allen L. M., Creaven P. J. (1975). Human pharmacokinetic model for isophosphamide (NSC-1097241).. Cancer Chemother Rep.

[OCR_00834] Allen L. M., Creaven P. J. (1972). In vitro activation of isophosphamide (NSC-109724), a new oxazaphosphorine, by rat liver microsomes.. Cancer Chemother Rep.

[OCR_00843] Anderson H., Hopwood P., Prendiville J., Radford J. A., Thatcher N., Ashcroft L. (1993). A randomised study of bolus vs continuous pump infusion of ifosfamide and doxorubicin with oral etoposide for small cell lung cancer.. Br J Cancer.

[OCR_00879] BROCK N., HOHORST H. J. (1963). UBER DIE AKTIVIERUNG VON CYCLOPHOSPHAMID IN VIVO UND IN VITRO. Arzneimittelforschung.

[OCR_00849] Boddy A. V., Idle J. R. (1992). Combined thin-layer chromatography-photography-densitometry for the quantification of ifosfamide and its principal metabolites in urine, cerebrospinal fluid and plasma.. J Chromatogr.

[OCR_00858] Boddy A. V., Yule S. M., Wyllie R., Price L., Pearson A. D., Idle J. R. (1995). Comparison of continuous infusion and bolus administration of ifosfamide in children.. Eur J Cancer.

[OCR_00868] Boddy A. V., Yule S. M., Wyllie R., Price L., Pearson A. D., Idle J. R. (1996). Intrasubject variation in children of ifosfamide pharmacokinetics and metabolism during repeated administration.. Cancer Chemother Pharmacol.

[OCR_00853] Boddy A. V., Yule S. M., Wyllie R., Price L., Pearson A. D., Idle J. R. (1993). Pharmacokinetics and metabolism of ifosfamide administered as a continuous infusion in children.. Cancer Res.

[OCR_00873] Brade W. P., Herdrich K., Varini M. (1985). Ifosfamide--pharmacology, safety and therapeutic potential.. Cancer Treat Rev.

[OCR_00877] Brock N. (1983). The oxazaphosphorines.. Cancer Treat Rev.

[OCR_00883] Creaven P. J., Allen L. M., Alford D. A., Cohen M. H. (1974). Clinical pharmacology of isophosphamide.. Clin Pharmacol Ther.

[OCR_00887] Hartley J. M., Hansen L., Harland S. J., Nicholson P. W., Pasini F., Souhami R. L. (1994). Metabolism of ifosfamide during a 3 day infusion.. Br J Cancer.

[OCR_00896] Kaijser G. P., Beijnen J. H., Bult A., Underberg W. J. (1994). Ifosfamide metabolism and pharmacokinetics (review).. Anticancer Res.

[OCR_00892] Kaijser G. P., Korst A., Beijnen J. H., Bult A., Underberg W. J. (1993). The analysis of ifosfamide and its metabolites (review).. Anticancer Res.

[OCR_00900] Klein H. O., Wickramanayake P. D., Christian E., Coerper C. (1984). Therapeutic effects of single-push or fractionated injections or continuous infusion of oxazaphosphorines (cyclophosphamide, ifosfamide, Asta Z 7557).. Cancer.

[OCR_00913] Lind M. J., Margison J. M., Cerny T., Thatcher N., Wilkinson P. M. (1989). Comparative pharmacokinetics and alkylating activity of fractionated intravenous and oral ifosfamide in patients with bronchogenic carcinoma.. Cancer Res.

[OCR_00920] Lind M. J., Margison J. M., Cerny T., Thatcher N., Wilkinson P. M. (1989). Prolongation of ifosfamide elimination half-life in obese patients due to altered drug distribution.. Cancer Chemother Pharmacol.

[OCR_00925] Lind M. J., Roberts H. L., Thatcher N., Idle J. R. (1990). The effect of route of administration and fractionation of dose on the metabolism of ifosfamide.. Cancer Chemother Pharmacol.

[OCR_00930] Morgan L. R., Harrison E. F., Hawke J. E., Hunter H. L., Costanzi J. J., Plotkin D., Tucker W. G., Worrall P. M. (1982). Toxicity of single- vs. fractionated-dose ifosfamide in non-small cell lung cancer: a multi-center study.. Semin Oncol.

[OCR_00936] Murray M., Butler A. M., Stupans I. (1994). Competitive inhibition of human liver microsomal cytochrome P450 3A-dependent steroid 6 beta-hydroxylation activity by cyclophosphamide and ifosfamide in vitro.. J Pharmacol Exp Ther.

[OCR_00942] Nelson R. L., Allen L. M., Creaven P. J. (1976). Pharmacokinetics of divided-dose ifosfamide.. Clin Pharmacol Ther.

[OCR_00946] Rodriguez V., Bodey G. P., Freireich E. J., McCredie K. B., McKelvey E. M., Tashima C. K. (1976). Reduction of ifosfamide toxicity using dose fractionation.. Cancer Res.

[OCR_00951] Ruzicka J. A., Ruenitz P. C. (1992). Cytochrome P-450-mediated N-dechloroethylation of cyclophosphamide and ifosfamide in the rat.. Drug Metab Dispos.

[OCR_00956] Wagner T. (1994). Ifosfamide clinical pharmacokinetics.. Clin Pharmacokinet.

[OCR_00957] Walker D., Flinois J. P., Monkman S. C., Beloc C., Boddy A. V., Cholerton S., Daly A. K., Lind M. J., Pearson A. D., Beaune P. H. (1994). Identification of the major human hepatic cytochrome P450 involved in activation and N-dechloroethylation of ifosfamide.. Biochem Pharmacol.

[OCR_00964] Weber G. F., Waxman D. J. (1993). Activation of the anti-cancer drug ifosphamide by rat liver microsomal P450 enzymes.. Biochem Pharmacol.

[OCR_00968] Yule S. M., Boddy A. V., Cole M., Price L., Wyllie R., Tasso M. J., Pearson A. D., Idle J. R. (1995). Cyclophosphamide metabolism in children.. Cancer Res.

